# Impact of “intensive parenting attitude” on children’s social competence via maternal parenting behavior

**DOI:** 10.3389/fpsyg.2024.1337531

**Published:** 2024-05-03

**Authors:** Sonoko Egami

**Affiliations:** Department of Developmental and Clinical Psychology, Shiraume Gakuen University, Kodaira, Japan

**Keywords:** intensive parenting attitude, intensive mothering, positive responsivity, involvement and monitoring, prosocial behavior, hyperactivity/inattention

## Abstract

“Intensive parenting” is a tendency to invest parents’ time, money, and energy in their child. This also includes some gender bias concerning a mother being the best person to primarily raise her children. Some psychology scholars have pointed out that this attitude causes much stress, anxiety, depression, and a sense of guilt among mothers. However, its effects on children have yet to be revealed using an extensive survey, and this indicates the need to investigate any possible impact of an intensive parenting attitude on children. The aim of this study was to elucidate a link between a maternal intensive parenting attitude and their children’s social competence through maternal parenting behavior. This was based on collecting data from 675 Japanese women who were mothers of preschoolers using the Japanese version of the Intensive Parenting Attitude Questionnaire, the Positive and Negative Parenting Scale, and the Strength and Difficulties Questionnaire. The results showed that the “Essentialism,” “Fulfillment,” and “Child-centered” components of intensive parenting attitude influenced the “involvement and monitoring” and “positive responsivity” of parenting behavior. Furthermore, these two parenting behaviors affected children’s prosocial behavior and hyperactivity/inattention. These findings suggest that an intensive parenting attitude has some impact on children as well as mothers, both positively and negatively, pointing to a serious effect on society.

## 1 Introduction

Motherhood brings numerous changes to women’s lives, including their appearance, inner hormonal balance, mental health, and perceptions (Hrdy, 2000). However, what may make a decisive difference is the people around and the society to which the mother belongs. Many scholars have suggested that the “myth of motherhood” has a considerable impact on people in some developed countries (e.g., United States, United Kingdom, and Japan)—the mother herself often embraces an ideal image of the “good mother.”

Intensive parenting is an ideology that mothers’ emotion, energy, time, and money should be focused on their children (Hays, 1996). It is suggested that this has been a major strategy of parenting in many developed countries. Furthermore, as the study of “intensive parenting” garnered increased attention, Liss et al. (2013) developed and validated a corresponding “intensive parenting attitude” scale. This questionnaire has five factors: Essentialism, the notion that women are inherently better at parenting than men and are exclusively given the role of child-rearing; Fulfillment, the belief that parenting should be fulfilling and a delight; Stimulation, the idea that children should be cognitively and intellectually stimulated by parents; Challenging, the belief that parenting is difficult and the most demanding job; and Child-centered, the notion that parents should prioritize the needs of the child above all else. Both mothers’ and fathers’ time spent with children has increased since the 1960s (Sayer et al., 2004). However, while both parents have shown changes in their attitudes, it is especially evident in mothers due to the influence of a separate ideological sphere (Cha, 2010). While social scientists have attributed these differences in parenting attitudes to factors such as parental financial status (Elliott et al., 2015), working status (Christopher, 2012), or educational background (Walls et al., 2014), there is also a notable prevalence of a strong belief in intensive parenting in developed countries (Bennet et al., 2012; Ishizuka, 2019). For instance, intensive parenting has been studied extensively in countries like the United States (Gunderson and Barrett, 2015), the United Kingdom (Cappellini et al., 2019), Canada (Wall, 2010), France (Loyal et al., 2017), Australia (Craig et al., 2014), and Japan (Egami, 2020).

In Japan, a high degree of intensive parenting attitude was found among mothers with preschool children, and especially they strongly embraced Essentialism (Egami, 2020). A great deal of research has been conducted on mother–child ties and maternal devotion to children in Japan (Kashiwagi, 1998). While recently Japanese women have changed to being more individualistic and working outside after childbearing, there is a strong belief in the myth of motherhood and ideal images of good mothers (Aono and Kashiwagi, 2011). What or whom could this affect? Egami (2005, 2007, 2013) showed the influence of the belief in “maternal love,” defined as unconditional maternal love for children, on mothers’ behavior toward children. In particular, the influence of this belief had a double-sided effect on maternal behaviors. Despite a background of gender disparity—for example, see the Global Gender Gap Index 2023 (World Economic Forum, 2023)—Egami (2017) stated that adherence to maternal love and devotion to their children is much stronger than endorsement of the gender division of labor. Since intensive parenting attitude encompasses five key factors, central to which is the belief that parents should prioritize their children above all and remain devoted to them, such attitudes could be the driving force behind Japanese mothers’ strong sense of child-rearing responsibility. Thus, it is crucial to investigate the impact of these attitudes on mothers in Japan where a high degree of intensive parenting attitude is prevalent among mothers of preschool children. In summary, since Japanese mothers might embrace the belief in maternal love and devotion to children regardless of socioeconomic status, educational background, and social support, it is invaluable to determine how strong such an intensive parenting attitude is for Japanese mothers generally, using a measure commonly used around the world.

### 1.1 Intensive parenting and its impact on mothers

Many scholars suggest that intensive parenting attitude harm maternal mental health and well-being (Wall, 2010; Liss et al., 2013; Rizzo et al., 2013; Meeussen and Van Laar, 2018; Prikhidko and Swank, 2018). Specifically, all factors of the intensive parenting attitude were positively correlated with separation anxiety in the Parental Investment in the Child Questionnaire (PIC) but Fulfillment was positively correlated with “delight” in the PIC and “satisfaction” in the Parenting Sense of Competence scale (Liss et al., 2013). Rizzo et al. (2013) found that Essentialism was negatively correlated with life satisfaction, and Challenging was positively correlated with depression and stress. Meeussen and Van Laar (2018) reported that mothers’ intensive mothering beliefs affected parental burnout via maternal gatekeeping behaviors. Based on in-depth interviews, Wall (2010) indicated that intensive parenting could increase maternal stress, exhaustion, anxiety, and guilt. Similarly, qualitative methods showed that unrealistic expectation derived from intensive mothering led mothers to struggle with meeting that demand, as a result, they felt a sense of guilt and self-blame when they could not achieve being an ideal mother (Prikhidko and Swank, 2018). In short, an intensive parenting attitude partly had a positive effect on the maternal psychological state (i.e., parental efficacy and delight); however, mostly it could damage maternal mental health and well-being.

Moreover, an intensive parenting attitude can affect maternal behavior, especially to preschoolers. Schiffrin et al. (2015) found that maternal intensive parenting attitude—especially those rooted in Essentialism, Stimulation, and Child-centered—were related to anticipatory problem-solving behavior indicative of overparenting as described by Segrin et al. (2012). This overparenting behavior was, in turn, associated with a higher likelihood of enrolling children in structured activities, including creative and physical ones. Fischer (2022) reported that five-year-olds’ parents who had a high degree of intensive parenting attitude showed higher probability of reading to their children more frequently. Also, Essentialism and Challenging of intensive parenting attitude were positively correlated with maternal “parent anger experience” and “parent anger expression” (Prikhidko and Swank, 2019). This study indicated that mothers who rated high on Essentialism and Challenging may be exhausted but have insufficient self-care because of a high degree of the responsibility for children. Then, they might become angry and finally blame their children. Furthermore, Egami (2020) conducted a comprehensive analysis of the intricate relationship between intensive parenting attitude and a spectrum of parenting behaviors (Ito et al., 2014), encompassing practices such as “positive responsivity,” fostering a “respect for will” (the child’s autonomy), active and diligent “involvement and monitoring,” alongside tendencies toward “overprotection,” the application of “harsh discipline,” and behavioral “inconsistency.” Consequently, every factor of intensive parenting attitude affected various maternal behaviors after controlling for social support. Apparently, Essentialism had a negative effect on positive responsivity. In contrast, Fulfillment had positive effects on both involvement and monitoring and positive responsivity. This result is consistent with some previous research (Liss et al., 2013). Interestingly, Stimulation positively affected positive responsivity, respect for will, and overprotection. According to the items related to Stimulation, mothers who embraced these beliefs tend to be education-minded parents. Although they may monitor their parenting behavior to ensure positive outcomes for their children, it is possible that they engage deeply in intensive parenting practices. As might be expected, Challenging was positively correlated with inconsistency and harsh discipline. This is because mothers being in a state of exhaustion will not have mild, stable, and consistent behavior toward their children. Surprisingly, being Child-centered affected more kinds of child-rearing behavior than any other factor. In particular, mothers who rated high on Child-centered had lower involvement and monitoring, overprotection, and harsh discipline, but higher respect for will. These results suggested that those who rated high on Child-centered might have tried to respect their children’s thought, seeking not to be intrusive toward their children’s feelings, and to have a warm attitude toward them.

In addition, there is some research on the effect of intensive parenting attitude, which suggested a relationship between intensive parenting attitude and maternal career ambitions (Meeussen and Van Laar, 2018) or partner relationships (Williamson et al., 2023). However, its effects on children have yet to be revealed using both extensive questionnaires and in-depth interviews. Intensive parenting attitude is less likely to directly relate to children’s outcomes since it is only the idea or belief of mothers. Still, there is a possibility of affecting children via the parenting behavior toward children. Since it is suggested that parenting behavior affects outcomes of child development, intensive parenting attitude could affect outcomes for children via maternal parenting behavior.

### 1.2 Parenting and child development

Many scholars and researchers have shown that parenting behavior can affect children’s behavior and developmental outcome. For example, Baumrind (1966, 1967, 1978, 1996, 2012) suggested that authoritative parenting could develop children’s self-control, positiveness, and friendly attitude. Authoritative parenting consists of inductive discipline, positive responsivity, respects for children’s will, and clear communication with children. Since then, there have been a growing number of studies in this field. Recently, Eti (2023) found that the authoritative parenting style and supportive beliefs about children’s emotions predicted children’s social skills.

Related to intensive parenting attitude, studies on overparenting and “concerted cultivation” have increased. Some scholars have pointed out that overparenting has the potential to lead to developmentally inappropriate parenting through excessive advice, problem-solving behavior, and provision of unnecessary assistance, combined with risk aversion (Segrin et al., 2012). This could manifest as a problem when a child reaches emerging or young adulthood, since individuals at that age need to develop autonomy and a sense of control themselves (Winner and Nicholson, 2018; Segrin and Flora, 2019; Hong and Cui, 2023). The effects of overparenting on young children, e.g., preschoolers or school-aged, are not yet clear except for studies by Bayer et al. (2006) and Gar and Hudson (2008). Although there is a paucity of research specifically focusing on the effects of overparenting in young children, the practice of concerted cultivation has been more thoroughly examined and is commonly studied within this age group.

Concerted cultivation is to actively foster children’s talents and skills through organized leisure activities and extensive reasoning behavior (Lareau, 2002). Concerted cultivation has been contrasted with “natural growth” (defined as providing the conditions under which children can grow but leaving leisure activities to children themselves and giving them clear directives) in some studies. Lareau (2002) stated that the predominant parenting style among middle- or upper-class families in the US is concerted cultivation, and that this style of parenting leads to an emerging sense of entitlement in the child. Although some scholars suggested that this type of parenting brought their children academic success (Carolan and Wasserman, 2015), others indicated that it may harm children’s mental health in particular during adolescence (Leung, 2020). However, the effects on a young child’s development have yet to be revealed.

There has been much research on the relationship between parenting (behavior or type) and child development in Japan. For example, Sugawara et al. (2002) found that a maternal warm attitude toward children predicted lower depression in school-aged children. In addition, in the study of Matsuoka et al. (2011), mothers’ positive rearing was negatively correlated with tendency for Attention-Deficit/Hyperactivity Disorder (ADHD), a neurobiological condition characterized by core symptoms of inattention, hyperactivity, and impulsivity (Chen et al., 2022), in preschool, elementary, and middle school children. As described, Japanese scholars implied that mothers’ positive parenting behaviors rather than a particular parenting behavior (i.e., overparenting) had a strong influence on their children. In their comprehensive meta-analysis, Ito et al. (2014) explored the multifaceted nature of parenting, initially categorizing behaviors into six distinct factors: involvement and monitoring, positive responsivity, respect for will, overprotection, inconsistency, and harsh discipline. Crucially, their study further distilled these factors into two overarching dimensions of parenting styles. The first three factors—involvement and monitoring, positive responsivity, and respect for will—were collectively identified as indicators of positive parenting behavior. In contrast, the latter three elements—overprotection, inconsistency, and harsh discipline—were found to typify negative parenting behavior. This bifurcation into positive and negative parenting behaviors offers a nuanced framework for understanding the complex dynamics inherent in parent–child interactions. In addition, they found that positive parenting behavior was correlated with school-aged children’s prosocial behavior, but negative parenting behavior was correlated with conduct problems of children. Moreover, Murayama et al. (2018) indicated that negative parenting behaviors were related to school-aged children’s experiences of bullying, including being the bully, the victim, and the bully-victim. Since the idea of second-order factors of parenting behavior is convincing evidence of parenting behavior in Japan, I used these scales and items applied to parenting behavior toward preschool children.

### 1.3 Research aims and hypotheses

This study examines the impact of intensive parenting attitude on child developmental outcomes through maternal parenting. First, the relationships of five factors of intensive parenting attitude, six factors of maternal parenting behavior, and five components of children’s outcomes were tested using correlational analysis to understand the overall relationship. Then structural equation modeling (SEM) was used to clarify the impact of intensive parenting attitude on both mothers and children. However, as stated before, intensive parenting attitude would not directly relate to children’s outcomes because the attitude is only the idea or belief of mothers. Therefore, I speculated that intensive parenting attitude indirectly influenced children’s outcome via maternal parenting behavior because Egami (2020) showed that intensive parenting attitude affected both positive and negative maternal parenting behaviors. In addition, a growing body of previous research, such as that by Eti (2023), suggested that maternal parenting behaviors had a strong effect on children.

As mentioned above, many previous studies suggested that Essentialism and Challenging of intensive parenting attitude have negative effects on maternal mental health and parenting behavior. In the study of Egami (2020), Essentialism was related to a low level of positive parenting behavior (e.g., positive responsivity), and Challenging had a relationship with high level of negative parenting behaviors (e.g., inconsistency and harsh discipline). In contrast, Fulfillment might increase maternal positive parenting behaviors (e.g., positive responsivity and involvement and monitoring). Finally, this implied that Child-centered and Stimulation were both positively and negatively correlated with maternal parenting behaviors. While Child-centered could decrease negative parenting behaviors (e.g., overprotection and harsh discipline), its high rating was also related to a low level of positive parenting behavior (e.g., involvement and monitoring). Stimulation might enhance maternal positive parenting behaviors (e.g., positive responsivity and respect for will); however, it could also increase negative parenting behavior (e.g., overprotection). Additionally, a number of scholars have stated that maternal parenting behavior does affect children, particularly their socioemotional development (e.g., Baumrind, 2012). Baumrind’s (1966, 1967, 1978, 1996, 2012) concept of authoritative parenting, often equated with positive parenting, is linked to children’s self-control, positiveness, and a friendly attitude. By contrast, maternal negative parenting behavior (i.e., harsh parenting) was correlated with the inability of children to regulate their emotions, as indicated by Chang et al. (2003).

**Figure 1:**
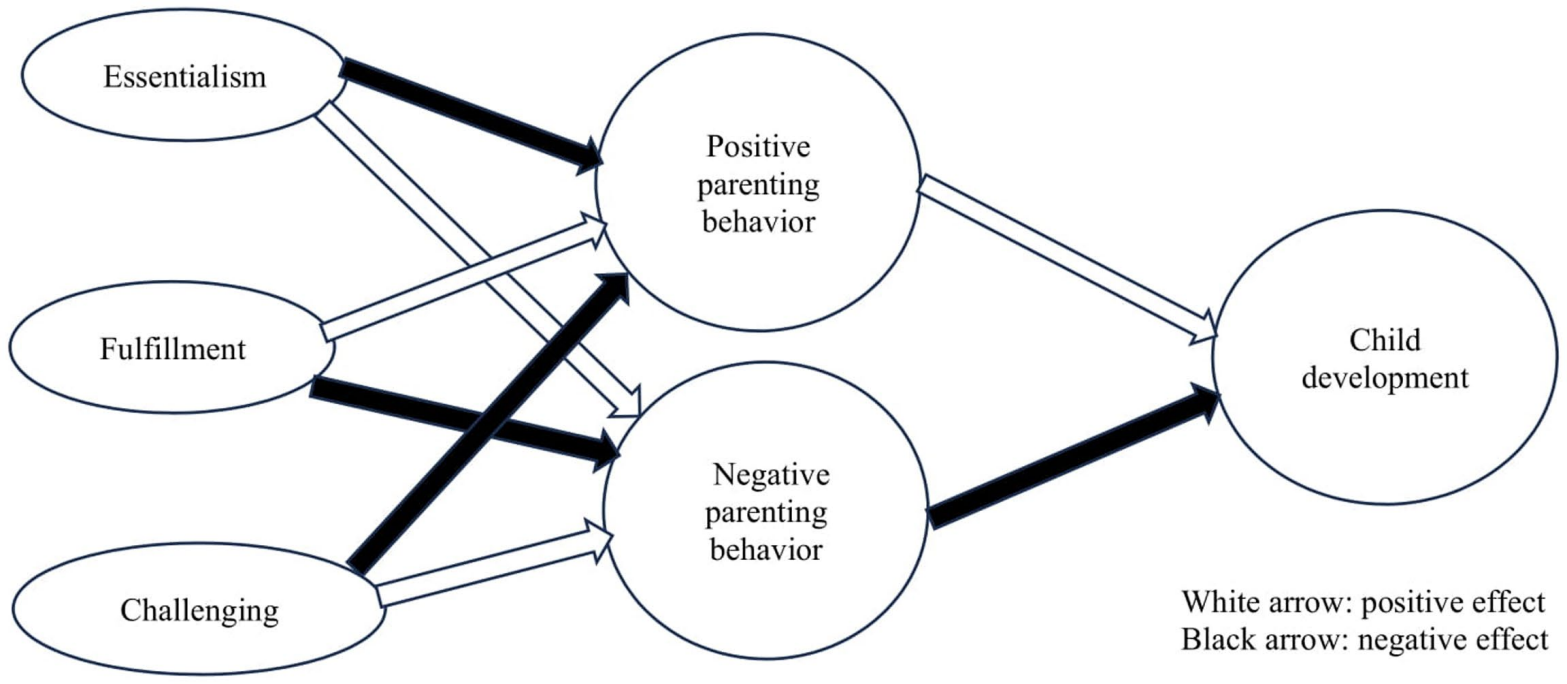
Hypothesized model of relations among intensive parenting attitude (excluding stimulation and child-centered), parenting behavior, and child development.

Therefore, I constructed the process model such that the impact of intensive parenting attitude would appear in maternal parenting behaviors, and these parenting behaviors might be related to children’s social outcomes. Specifically, the goal of this study was to test four hypotheses (see Figure 1):

*H1*: Intensive parenting attitude will affect children’s social development only via parenting behavior.

*H2*: Essentialism and Challenging will increase negative parenting behavior and decrease positive parenting behavior. Then, these will lead to poor social development in children.

*H3*: Fulfillment will increase positive parenting behavior and decrease negative parenting behavior. Then, these will be positively related to social development in children.

*H4*: Child-centered and Stimulation will affect both positive and negative parenting behavior, so having double-sided effects on children.

To effectively test the four hypotheses, controlling for the age of the children was essential, as this can influence maternal parenting behaviors and the outcomes for these children. For instance, within the preschool age group, parents may differentiate their behaviors between 2- and 6-year-olds, especially for monitoring, protecting, controlling, and discipline (Peterson et al., 1993). In addition, the score of children’s outcomes can vary just due to their age (Croft et al., 2015). In sum, this study aims to clarify the impact of intensive parenting attitude on not only mothers but also children through verification of the four hypotheses. Egami (2020) stated that Japanese mothers’ tendency of intensive parenting attitude affected their parenting behavior toward their children. So, could the impact of intensive parenting attitude spill over to the children? If so, how does it relate to them? To what extent does it correlate with them? Which part of intensive parenting attitudes could influence them? Revealing these could show that the impact of intensive parenting attitude is significant in society.

## 2 Materials and methods

### 2.1 Procedure

Respondents were asked to answer the questionnaire anonymously via an Internet research company (Macromill Inc.), who ensured its compliance with the privacy policy established by the Japan Marketing Research Association. To measure the impact of intensive parenting attitude on parenting behavior over time, the survey was divided over two time periods: November 2017 and April 2018. Background variables and the Japanese version of the Intensive Parenting Attitude Questionnaire (J-IPAQ) were collected during the first time period, November 2017. The Positive and Negative Parenting Scale (PNPS) and the Strength and Difficulties Questionnaire (SDQ) were measured at the second period.

### 2.2 Participants

Participants were 675 Japanese mothers aged 22–48 years (mean 34.7, standard deviation 5.0). They had at least one preschool-age child (aged 1 years 6 months to 6 years 10 months), thereby holding relatively intense child-rearing responsibility. Most of them were housewives (59.1%), and the rest were full-time workers (13.8%), part-time workers (20.0%), or freelancers (7.1%). The majority were married (97.3%). Household annual income level was classed from 1 (less than ¥2 million) to 9 (more than ¥20 million). Class 3 (¥4 million to less than ¥6 million) was the majority (29.5%), followed by class 2 (¥2 million to less than ¥4 million; 18.7%) and class 4 (¥6 million to less than ¥8 million; 13.3%). Average household annual income was about ¥5.7 million in Japan at that time, so most of them fitted into the average income category. About one-quarter of them (23.4%) had at least a high school education, 35.7% graduated professional training college or junior college, and 36.9% of them had a bachelor’s degree. The length of education was 9–21 years (mean 14.2, standard deviation 1.77).

### 2.3 Measures

#### 2.3.1 J-IPAQ

Egami (2020) constructed a Japanese version of the intensive parenting attitude questionnaire, and both validity and reliability were confirmed. The original IPAQ of Liss et al. (2013) included five categories: Essentialism, Fulfillment, Stimulation, Challenging, and Child-centered. Essentialism, in the context of motherhood, posits that mothers have a natural and exclusive role in raising their children. Fulfillment is the notion that child-rearing always brings joy and rewards for parents. Stimulation is the idea that parents must develop their children’s intellectual ability. Challenging refers to the difficulty, exhaustion, and tiredness accompanying parenting. Child-centered is the belief that children must be the center of parents’ lives and children’s needs should be prioritized before anything else. The IPAQ includes 25 items; however, J-IPAQ consists of 20 items (Egami, 2020). The J-IPAQ is rated from 1 (strongly disagree) to 6 (strongly agree), the higher the score, the greater the degree of each dimension of intensive parenting attitude. Cronbach alpha in this study was 0.73 for Essentialism (six items), 0.75 for Fulfillment (three items), 0.57 for Stimulation (four items), 0.63 for Challenging (four items), and 0.68 for Child-centered (three items).

#### 2.3.2 PNPS

Ito et al. (2014) constructed the PNPS, which consists of 35 items, divided into involvement and monitoring, positive responsivity, respect for will, overprotection, inconsistency, and harsh discipline. The PNPS is rated from 1 (not at all) to 5 (extremely). Positive parenting behavior includes involvement and monitoring, positive responsivity, and respect for will. Negative parenting behavior contains overprotection, inconsistency, and harsh discipline. The higher the score, the greater the degree of each group of parenting behavior. This study changed PNPS item expression “school” to “preschool, nursery, or kindergarten” because of the children’s age (the creator of this scale gave permission). The original scale has 35 items, eight items were eliminated from this study after confirmatory factor analysis (Egami, 2020). Cronbach alpha was 0.84 for involvement and monitoring (six items), 0.82 for positive responsivity (five items), 0.61 for respect for will (four items), 0.62 for overprotection (three items), 0.81 for inconsistency (three items), and 0.85 for harsh discipline (six items) in this study.

#### 2.3.3 SDQ

The SDQ is well-known scale measuring psychological attributes of children (Goodman, 1997). This study used the Japanese version of SDQ (for the parents of 2–4-year-olds) based on Matsuishi et al. (2008). It contains 25 items divided into five categories: “emotional symptoms,” “conduct problems,” “hyperactivity/inattention,” “peer relationship problems,” and “prosocial behavior.” The SDQ is rated from 0 (not true) to 2 (certainly true). The higher the score, the greater the degree of each dimension of psychological attributes. Cronbach alpha in this study was 0.80 for prosocial behavior (five items), 0.67 for hyperactivity/inattention (five items), 0.62 for emotional symptoms (five items), 0.49 for conduct problems (five items), and 0.49 for peer relationship problems (five items).

## 3 Results

Descriptive statistics are shown in Table 1. Stimulation, Challenging, respect for will, overprotection, emotional symptoms, conduct problems, and peer relationship problems were not used in the following analysis because of low internal consistency (Cronbach*α* < 0.65). The factors’ scores for J-IPAQ and PNPS were averaged by the number of items, but were summed up for the contained items in SDQ. Descriptive statistics and correlation analysis were performed using IBM SPSS Statistics (ver. 27.0) and structural equation modeling (SEM) analysis using Amos (ver. 28.0).

**Table 1:** Descriptive statistics of the main variables (*N* = 675).

	*M*	*SD*	Possible range	Actual range
Essentialism	3.69	0.78	1–6	1.17–6
Fulfillment	4.35	0.89	1–6	1–6
Child-centered	3.93	0.81	1–6	1–6
Involvement and monitoring	3.04	0.97	1–5	1–5
Positive responsivity	4.25	0.59	1–5	1.20–5
Inconsistency	2.97	0.71	1–5	1–5
Harsh discipline	2.79	0.67	1–5	1–5
Prosocial behavior	5.50	2.43	0–10	0–10
Hyperactivity/inattention	3.95	2.22	0–10	0–10

### 3.1 Correlation analysis

Correlations between all measures are shown in Table 2. Some correlation relationships appeared but correlation coefficients were relatively low. Most factors of intensive parenting attitude were correlated with parenting behavior. While Essentialism was positively correlated with the negative parenting behavior, both Fulfillment and Child-centered were negatively correlated with such behavior and positively correlated with positive parenting behavior. As expected, the factors concerning intensive parenting attitude were rarely related with children’s outcomes. Still, Essentialism was positively correlated with hyperactivity/inattention, and Fulfillment was positively correlated with prosocial behavior of children. Then, most maternal parenting behaviors had a connection with children’s outcomes. My research indicates a distinct relationship between parenting behaviors and child outcomes. Positive parenting, marked by involvement and monitoring or positive responsivity, correlates with beneficial outcomes in children, such as improved social skills and lower behavioral problems. This suggests that positive parenting actively fosters a nurturing environment crucial for children’s healthy development. On the other hand, negative parenting behaviors, including harsh discipline or inconsistency, are linked to adverse child outcomes, such as behavioral problems and lower prosocial behavior. Importantly, this study’s findings highlight that negative parenting does not inversely contribute (i.e., by its absence) to positive child outcomes. This distinction underscores that positive child development is more directly a result of positive parenting practices, rather than merely the absence of negative ones. These insights affirm the critical role of positive parenting in promoting not just the avoidance of harm, but in actively supporting comprehensive child development.

**Table 2:** Correlations between the measures (*N* = 675).

	1	2	3	4	5	6	7	8
Essentialism								
Fulfillment	0.14^***^							
Child-centered	0.28^***^	0.46^***^						
Involvement and monitoring	−0.06	0.14^***^	−0.01					
Positive responsivity	−0.07	0.35^***^	0.23^***^	0.17^***^				
Inconsistency	0.13^**^	−0.08^*^	−0.02	−0.02	−0.22^***^			
Harsh discipline	0.10^*^	−0.13^**^	−0.12^**^	0.03	−0.28^***^	0.69^***^		
Prosocial behavior	−0.02	0.21^***^	0.05	0.51^***^	0.26^***^	−0.06	−0.06	
Hyperactivity/inattention	0.11^**^	−0.10^**^	0.06	−0.30^***^	−0.22^***^	0.12^**^	0.18^***^	−0.42^***^

^***^p < 0.001, ^**^p < 0.01, ^*^p < 0.05.

### 3.2 SEM analysis

**Figure 2:**
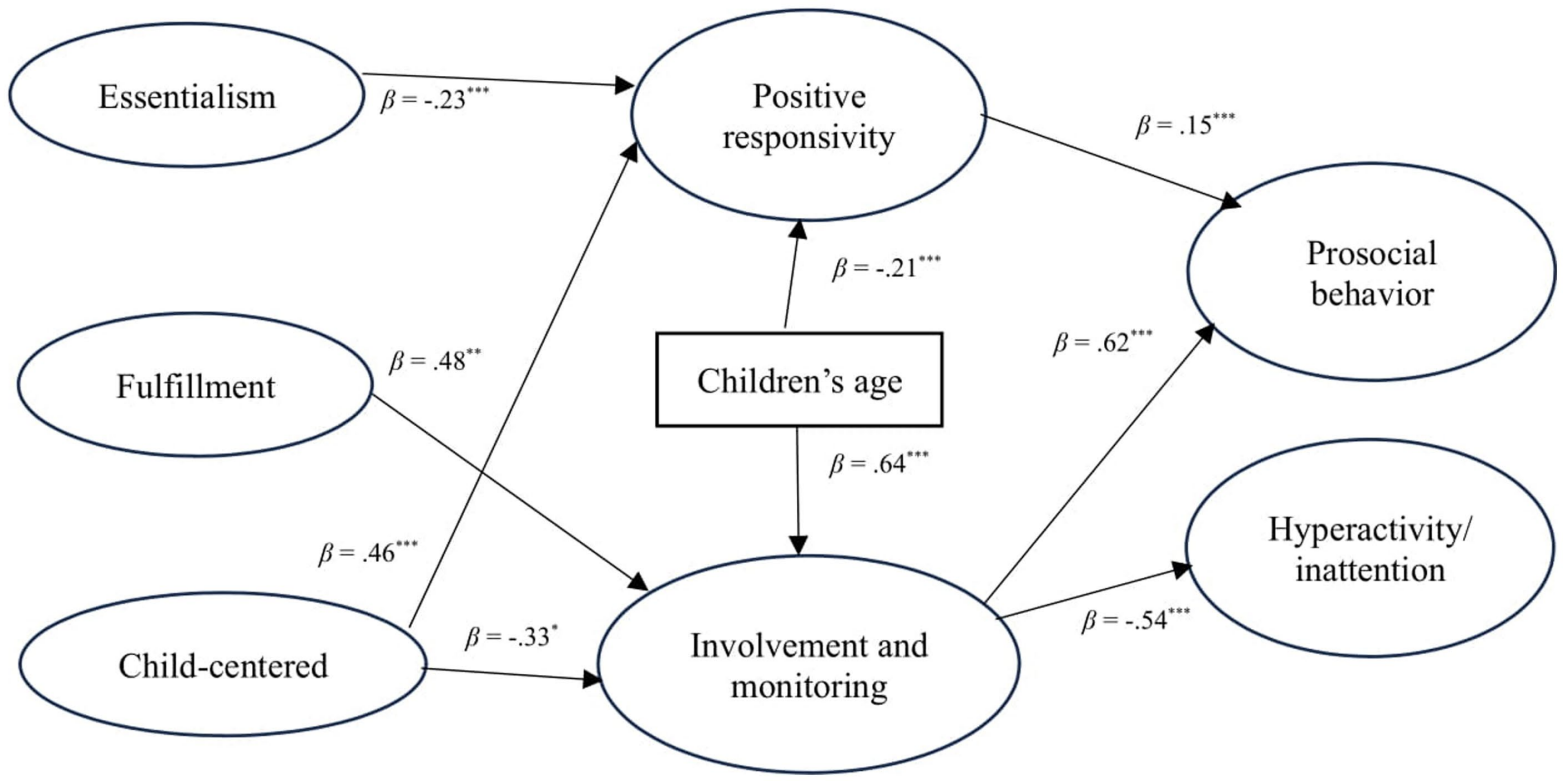
Graphical overview of significant paths in the final model (^***^p < 0.001, ^**^p < 0.01, ^*^p < 0.05).

The theoretical model proposed in this study, accounting for the influence of children’s age on both parenting behaviors and children’s outcomes, was rigorously tested using SEM (Tables 3, 4 and Figure 2). However, the analysis yielded an implausible score, indicating that the model did not accurately reflect the data (Model 1). This outcome suggests that the theoretical framework, as constructed, may not adequately capture the complexities of the relationships between intensive parenting attitude, maternal parenting behaviors, and children’s developmental outcomes. Further refinement and testing of the model are necessary to develop a more accurate representation of these dynamics. Therefore, inconsistency and harsh discipline were eliminated since their standardized coefficients yielded 1 (Model 2). Next, the non-significant paths were deleted (Model 3). Then, for fixed parameters, some covariances were added (Model 4). After confirming validity and reliability of all factors (Table 4), some items were removed because they caused the score of AVE and CR to decrease. Finally, the proposed model (as shown in Figure 2) fitted the data reasonably well: *χ*^2^(409) = 653.14 (*p* < 0.001), GFI = 0.94, AGFI = 0.93, CFI = 0.96, RMSEA = 0.03, AIC = 827.14, CAIC = 1306.92. Due to the large sample size of the study, the chi-square score was significant. Based on correlation coefficients in Table 2, multicollinearity was not found among scales. All items’ coefficients and the score of AVE (average variance extracted; validity score) and CR (composite reliability; reliability score) are shown in Table 4. Fornell and Larcker (1981) suggest that the criteria (AVE ≧ 0.4, CR ≧ 0.6) are desirable; however, when AVE is near 0.4, the scale has validity and reliability, if CR is over 0.6. Thus, most scales confirmed both validity and reliability except for Child-centered. Still, the proposed model including Child-centered was accepted since the factor’s Cronbach *α* was relatively high (*α* = 0.68) despite including only three items. Additionally, Child-centered in J-IPAQ was confirmed in Egami (2020), which used a similar sample to that for this study and the coefficient scores in these data were all significant.

**Table 3:** The goodness-of-fit score in all models (*N* = 675).

Model	Chi-square	GFI	AGFI	CFI	RMSEA	AIC	CAIC
Model 1	1995.06^***^	0.87	0.86	0.88	0.05	2223.06	2851.74
Model 2	1291.20^***^	0.90	0.88	0.88	0.05	1457.20	1914.92
Model 3	1010.81^***^	0.92	0.90	0.92	0.04	1190.81	1687.13
Model 4	772.40^***^	0.93	0.92	0.95	0.03	956.40	1463.75
The proposed model	653.14^***^	0.94	0.93	0.96	0.03	827.14	1306.92

***p < 0.001.

**Table 4:** List of all item’s coefficients and the score of AVE and CR (*N* = 675).

Factor	Item number	Standardized coefficient	AVE	CR	Factor	Item number	Standardized coefficient	AVE	CR	Factor	Item number	Standardized coefficient	AVE	CR
Essentialism	2	0.53^***^	0.38	0.74	Involvement and monitoring	6	0.59^***^	0.43	0.82	Prosocial behavior	1	0.58^***^	0.38	0.75
	4	0.40^***^				10	0.82^***^				4	0.58^***^		
	12	0.67^***^				19	0.65^***^				9	0.71^***^		
	17	0.74^***^				20	0.60^***^				17	0.62^***^		
	20	0.67^***^				24	0.57^***^				20	0.58^***^		
Fulfillment	7	0.72^***^	0.48	0.74		29	0.68^***^							
	14	0.72^***^			Positive responsivity	11	0.63^***^	0.48	0.82	Hyperactivity/ inattention	2	0.52^***^	0.40	0.66
	18	0.65^***^				13	0.73^***^				21	0.64^***^		
Child-centered	11	0.47^***^	0.25	0.49		18	0.73^***^				25	0.72^***^		
	19	0.64^***^				27	0.68^***^							
	24	0.36^***^				35	0.70^***^							

^***^p < 0.001.

Figure 2 presents relationships between intensive parenting attitude and children’s outcomes through parenting behaviors as well the standardized coefficient of each path. As mentioned above, Hypothesis 1 is that intensive parenting attitude has no direct effect on children’s outcomes; nonetheless, the analysis confirmed an indirect effect of parenting behavior on children’s outcomes, thereby substantiating the hypothesis. Hypotheses 2 and 3 that Essentialism and Challenging have negative impacts on children’s outcomes through parenting behavior, and that Fulfillment has positive impacts, were both partially supported. Moreover, Hypothesis 4 that Stimulation and Child-centered would have both positive and negative effects on children’s outcomes via parenting behavior was partly supported.

The more Essentialism mothers had, the less positive responsivity they exhibited (*β* = −0.23) (Figure 2). Consequently, less positive responsivity was associated with reduced prosocial behavior in children (*β* = 0.15). The more Fulfillment mothers experienced, the greater their involvement and monitoring (*β* = 0.48). Then parenting behavior could increase prosocial behavior (*β* = 0.62) and decrease hyperactivity/inattention (*β* = −0.54) of children. Finally, the more Child-centered they were, the less involvement and monitoring they exhibited (*β* = −0.33) yet they displayed more positive responsivity (*β* = 0.46). Through these path lines, being Child-centered had double-edged impacts on children’s outcomes.

## 4 Discussion

This study examined the impact of intensive parenting attitude on children’s social developmental outcomes via maternal parenting behavior. In summary, most of the hypotheses were supported to some degree. Schiffrin et al. (2015) found that intensive parenting attitude was related to anticipatory problem-solving behavior and children’s gross motor skills through enrollment in structured activities including creative and physical activities. However, their study was limited to only one parenting behavior and its effect was seen only in the outcomes of gross motor skill of children. In contrast, Egami (2020) suggested that intensive parenting attitude affected maternal parenting behavior; however, its influence on children was not examined. Therefore, the results of this study, including correlations, are worthy of a closer look.

First, Japanese mothers embraced Essentialism at a high rate (Egami, 2020). Given that Essentialism was positively correlated with negative parenting behaviors (i.e., inconsistency and harsh discipline), there is a need to investigate the risk of this belief to both mothers and children in Japan. As suggested by Rizzo et al. (2013) and others, Essentialism could harm not only mothers but also their children. Second, Fulfillment was positively related with positive parenting behaviors (i.e., involvement and monitoring, and positive responsivity) and negatively with negative parenting behaviors. Because this result seems consistent with that of Liss et al. (2013), mothers’ parental efficacy and delight in parenting could lead to their positive parenting behavior. Finally, being Child-centered was related to parenting behaviors (positive responsivity and harsh discipline). Correlation analysis revealed the positiveness of Child-centered attitude; however, SEM analysis showed that this belief had double-sided effects. Although Schiffrin et al. (2015) indicated that Child-centered attitude might affect anticipatory problem-solving behavior, Egami (2020) found that it had a negative relationship with overprotection which was closely related to anticipatory problem-solving behavior. Moreover, while being Child-centered was correlated with low life satisfaction of mothers in the study of Rizzo et al. (2013), it was correlated with parental delight and efficacy for Liss et al. (2013). Probably, Child-centered belief can have both positive and negative aspects for mothers, and might depend on the culture and situation surrounding them.

As mentioned above, there were many patterns of correlations between intensive parenting attitude and maternal parenting behavior, however, correlations between intensive parenting attitude and child outcomes were rarely seen. While measurements of intensive parenting attitude were conducted in April 2018, social development of children was obtained in November 2017. Given this timeline, “intensive parenting attitude” can be considered a potential predictor of the perception of social development in children. This may indicate that the positive view of child-rearing leads to a positive bias on child development. Conversely, Essentialism tends to have a negative effect on child development and to indicate mothers’ tiredness (Meeussen and Van Laar, 2018). The correlation pattern of maternal parenting behavior and child outcomes was the same as found by Ito et al. (2014), other than the relationship between negative parenting behavior and children’s positive outcomes. Since these data were collected at the same time, children’s outcomes might affect maternal behavior, and vice versa.

Although the correlational analysis revealed numerous relationships among variables, only a subset of these correlations was substantiated by the SEM analysis. The results indicated that Essentialism diminished the capacity of mothers to engage warmly with their children, which negatively impacted maternal positive responsivity and consequently reduced children’s prosocial behavior. This aligns with research suggesting that Essentialism can foster a negative mindset in mothers (Liss et al., 2013; Rizzo et al., 2013; Meeussen and Van Laar, 2018), thereby impairing their ability to interact positively with their children. In Japan, Egami (2005, 2007) showed that belief in maternal love negatively influenced mothers’ emotional regulation and expression toward children according to the situation surrounding mothers. Therefore, Essentialism damaged not only mothers’ mental health but also that of young children. Next, as expected, Fulfillment increased involvement and monitoring and positively influenced children’s prosocial behavior and negatively affected hyperactivity/inattention. According to Liss et al. (2013), Fulfillment can lead to maternal parenting efficacy and delight. Thus, mothers who embrace Fulfillment pour their energy into parenting behavior and engage positively with their children. Lastly, this study showed that Child-centered attitude had a double-sided effect on positive parenting behavior—it increased positive responsivity but decreased involvement and monitoring. This indicated that Child-centered attitude had both positive and negative impacts on children’s social development outcomes. As mentioned above, being Child-centered had a double-sided effect in some previous studies. Interestingly, Egami (2020) noted that Child-centered was negatively related to involvement and monitoring and overprotection; however, Schiffrin et al. (2015) showed that it had a positive relationship with anticipated problem-solving behavior. Similarly, while Child-centered attitude was negatively correlated with maternal life satisfaction (Rizzo et al., 2013), it was positively related to delight and efficacy as a parent (Liss et al., 2013). The belief of “for the sake of children” (i.e., a child centered approach) might make parents think from a child’s point of view. This idea can work differently under a wide variety of circumstances and cultures. In Japan, this may lead to more lenient parenting behavior (e.g., a natural growth strategy) as well as positive attitudes toward children. The findings suggest that an intensive parenting attitude has both positive and negative effects on mothers and children alike, pointing to potentially far-reaching implications for our society at large.

### 4.1 Conclusion

Hypothesis 1 was supported and Hypotheses 2, 3, and 4 were partially supported. Therefore, intensive parenting attitude had some effects on children as well as mothers. At the same time, this had a double-sided impact. Some factors of intensive parenting attitude might be related to parenting efficacy and positive maternal feelings related to child-rearing (Liss et al., 2013; Egami, 2020), and others could be connected to less positive responses toward children through maternal stress and tiredness. The “parenthood paradox,” as mentioned in Rizzo et al. (2013), reflects a unique dilemma in which maternal efforts to achieve perfection in parenting can ironically lead to increased stress and negative attitudes toward their children. This phenomenon underscores the unintended consequences of intensive parenting, potentially impacting a child’s social and emotional development. It highlights the need for a balance between high parental aspirations and realistic expectations, suggesting a shift in societal attitudes toward a more compassionate and feasible approach to parenting.

### 4.2 Limitations and future directions

This study revealed the impact of intensive parenting attitude on child development via maternal parenting behavior. However, the study has some limitations. First, the data of maternal parenting behavior and children’s developmental outcomes were collected at the same time. Therefore, it is impossible to establish a clear causal relationship between maternal parenting behavior and outcomes for children. This underscores the need for a longitudinal research approach. Second, the data for this study were derived from mothers’ self-reports only; however, mixed methods are required to produce more solid data. For example, in-depth interviews of mothers or observations of mother–child interactions are needed for research on intensive parenting attitude and maternal parenting behaviors. Third, this study utilized the parent-rated version of the SDQ to assess child behavior. While this version effectively captures the child’s behavior at home, it is important to note that it may not fully represent their behavior in other environments, such as in the educational settings. Typically, the SDQ is also available in a teacher-rated version, which can provide complementary insights into the child’s behavior across different settings. The absence of the teacher-rated SDQ in this study could be considered a limitation, as it restricts the scope of behavioral assessment in the home. Therefore, the results should be interpreted with caution, especially when generalizing about the child’s overall behavior or the impact of intensive parenting attitude. Fourth, this study’s analysis on intensive parenting attitude is limited to three factors because of the low reliability of the other two factors (Stimulation and Challenging). In addition, the low AVE and CR scores of Child-centered require further consideration and careful interpretation. Child-centered in this study might be labeled as a provisional factor, and there is a need for further replication. Future research on intensive parenting attitude should examine how all five factors of intensive parenting attitude affect maternal mental parenting behavior, especially negative parenting behaviors and children’s social outcomes. Through these research plans, the impact of intensive parenting attitude would give strong messages to our society, which includes mothers and children. Furthermore, there has been little research on intensive parenting attitude in Asia. Convincing research findings are needed to offer extensive and effective support for child-rearing, especially in Asian countries, including Japan.

## Data availability statement

The raw data supporting the conclusions of this article will be made available by the author, without undue reservation.

## Ethics statement

The studies involving humans were approved by the Ethics Committee in the Faculty of Education, Ehime University. The studies were conducted in accordance with the local legislation and institutional requirements. The participants provided their written informed consent to participate in this study.

## Author contributions

SE: Writing – original draft.
